# Cancer patients on Twitter: a novel patient community on social media

**DOI:** 10.1186/1756-0500-5-699

**Published:** 2012-12-27

**Authors:** Yuya Sugawara, Hiroto Narimatsu, Atsushi Hozawa, Li Shao, Katsumi Otani, Akira Fukao

**Affiliations:** 1 Department of Medical Informatics, Graduate School of Medical Science, Yamagata University, Yamagata, Japan; 2 Advanced Molecular Epidemiology Research Institute, Faculty of Medicine, Yamagata University, Yamagata, Japan; 3 Department of Public Health, Yamagata University Graduate School of Medicine, Yamagata, Japan

**Keywords:** Breast cancer, Breast neoplasms, Internet, Leukemia, Social media, Twitter messaging, Web 2.0

## Abstract

**Background:**

Patients increasingly turn to the Internet for information on medical conditions, including clinical news and treatment options. In recent years, an online patient community has arisen alongside the rapidly expanding world of social media, or “Web 2.0.” Twitter provides real-time dissemination of news, information, personal accounts and other details via a highly interactive form of social media, and has become an important online tool for patients. This medium is now considered to play an important role in the modern social community of online, “wired” cancer patients.

**Results:**

Fifty-one highly influential “power accounts” belonging to cancer patients were extracted from a dataset of 731 Twitter accounts with cancer terminology in their profiles. In accordance with previously established methodology, “power accounts” were defined as those Twitter accounts with 500 or more followers. We extracted data on the cancer patient (female) with the most followers to study the specific relationships that existed between the user and her followers, and found that the majority of the examined tweets focused on greetings, treatment discussions, and other instances of psychological support. These findings went against our hypothesis that cancer patients’ tweets would be centered on the dissemination of medical information and similar “newsy” details.

**Conclusions:**

At present, there exists a rapidly evolving network of cancer patients engaged in information exchange via Twitter. This network is valuable in the sharing of psychological support among the cancer community.

## Background

Health-focused websites have become an increasingly valuable information source for cancer patients in recent years, with such patients seeking details about treatment options for their specific condition as well as about general cancer information [1-3]. These websites provide a means of communication for patients and their families that is more convenient and less expensive than that provided by traditional face-to-face patient-serving health organizations [2]. In a previous study, we suggested that patient-authored web logs (or “blogs”) represent a unique form of information delivery as they provide useful personal insights about cancer treatment that are unlike the information often conveyed by healthcare providers through face-to-face interactions and standard media [1]. Such patient-centric sites are also becoming a valuable source of personalized health information for the increasingly “wired” cancer-patient communities across the globe.

Attendant to the continuing rise in social media (“Web 2.0”) participation and the resulting proliferation of user-generated online content, the public can thus potentially play a larger role in all stages of knowledge translation, including information generation, filtering and amplification. As with the Internet itself, social media outlets run the gamut of just about every imaginable scope and size, with Twitter, a free social-networking and micro-blogging service launched in 2006, taking the lead as a method of disseminating exceptionally brief online messages to a potentially global audience; Twitter enables its millions of users to send and read each other’s “tweets,” or short messages limited to 140 characters, with the users themselves determining whether their tweets can be read by the general public or restricted to preselected “followers.” Followers of a specific Twitter user can view or respond to tweets online or via smart phones and other handheld devices, allowing for a nearly instantaneous dialogue between the user and his or her followers. The service has more than 190 million registered users worldwide and processes about 55 million tweets per day [4]. The Twitter service started in Japan in 2008; at present, there are more than 10.2 million active Twitter accounts registered in the country [5].

A recent health-focused analysis of the American “Twitter stream” revealed that a substantial proportion of tweets contain general chatter, user-to-user conversations that are only of interest to the parties involved, links to interesting pieces of news or self-promotion or unwanted “junk” messages (i.e., spam) [4]. Yet despite its high level of noise, the Twitter stream does contain useful information. Many recent news events or scientific issues have been documented and discussed via Twitter directly from users at the site in real time [6].

As tweets are often sent on location via smart phones and other handheld platforms,they convey more immediacy with interactivity than other websites or blogs [4]. In addition, healthcare providers and medical researchers are increasingly using Twitter for a variety of purposes related to patient care and treatment, including sharing clinical news with patients and discussing case studies with fellow physicians [7-11]. A recent *JAMA* letter showed that physicians frequently use Twitter to share medical information, with nearly half of the studied tweets being devoted to the discussion of health topics; the authors found that physicians’ rapid and timely dissemination of such information via Twitter could potentially positively influence public health in a variety of ways [12].

Recent research has also shown that Twitter may also be a useful medium for patients, who use Twitter to exchange medical information and discuss various aspects of their individual illness; although detailed information about patients’ use of Twitter for such purposes has yet to be fully studied, it has been shown that some patients with breast cancer, chronic kidney disease, diabetes and inflammatory bowel disease have used Twitter for the purpose of sharing information about these conditions [13-18].

Twitter is an interactive, real-time medium that can be used at a relatively low cost in terms of users’ initial and ongoing monetary investment and in the time, effort and expertise required for use. Furthermore, as has been described above, Twitter has been effectively used in recent years for the dissemination of medical news and advice, as well as the delivery of “personal stories” related to a number of health topics. As a result, Twitter can be considered to have the potential to play an important role in modern social communities, including online communities consisting of “wired” cancer patients. However, the research conducted to date regarding the role of social media in influencing cancer patients remains very limited. In this study, we examine recent Twitter usage in Japan and evaluate its role in the lives of today’s “wired” cancer patients.

## Methods

### Search of cancer Patients’ Twitter accounts

A search was conducted of every publicly available user profile on Twitter in Japan. We began this search by reviewing all user accounts in which the names of cancers were described in the user’s Twitter profile. The cancer names used in our search were obtained in accordance with the Foundation for Promotion of Cancer Research’s 2010 report on Japanese cancer rates [19]. The terms searched were: breast cancer, leukemia, colon cancer, rectal cancer, colorectal cancer, cancers of the uterus, malignant lymphoma, brain tumor, stomach cancer, lung cancer, thyroid cancer, ovary cancer, kidney cancer, prostate cancer, esophagus cancer, bladder cancer, liver cancer, oral cancer, pharyngeal cancer, gallbladder cancer, cholangiocarcinoma, laryngeal cancer, skin cancer and multiple myeloma. These names were searched using both the Japanese Katakana writing system and Chinese characters.

The website used for the profile search was the “16 (one-six) Profile Search β Version for Twitter” [20], which enabled us to search, in addition to users’ Twitter profiles, the number of follows, followers, tweets, lists, registered dates and last-posted dates. The search was conducted over a total of 5 days in the spring and summer of 2011: March 27, 28 and 29; April 3; and July 12. Following the methodology used by Chretien et al. (2011) [12], we then extracted from our dataset of cancer profiles only those user accounts that had 500 or more followers; we considered these to be “power accounts,” as they had each developed a relatively robust Twitter following.

**Figure 1:**
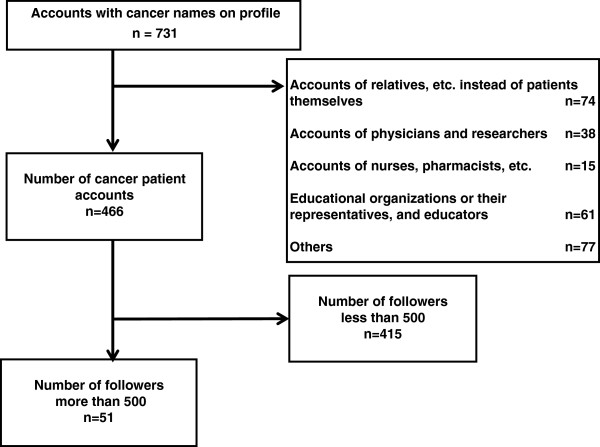
Extraction of cancer patient accounts.

Our search of Japanese Twitter profiles that included the cancer terminology noted above yielded a total of 731 user accounts, of which 466 profiles belonged to cancer patients and were included in our initial review. The remaining 265 cancer profiles were excluded from our initial analysis because they belonged to persons and organizations who were not patients themselves (Figure 1).

**Figure 2:**
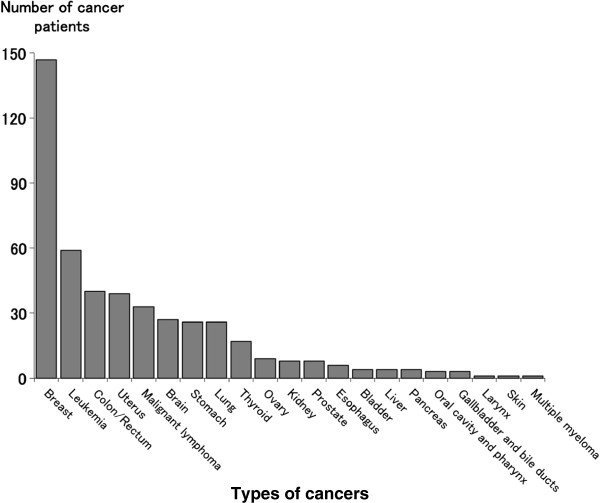
**Number of accounts by type of cancer.** Number of accounts of self-identified cancer patients, with cancer names described in their profiles. In the case of multiple cancers, each cancer was counted.

Among the initial 731 user accounts that included cancer terminology, breast cancer was listed in user profiles most frequently (n=147), followed by leukemia (n=59), colon/rectal/colorectal cancer (n=40) and uterine cancer (n=39). Those patients who listed multiple cancers in their Twitter profiles were counted separately (Figure 2).

Fifty-two Twitter accounts with the relevant cancer descriptions in their profiles met the criterion established by Chretien et al. (2011) [12] required for being “power accounts and were considered by us to be influential accounts because of their wide reach. (The account with the most followers belonged to a comedian with breast cancer; because of the user’s celebrity status, the difficulty of adequately tracking tweets between the user and her followers and the fact that the vast majority of the user’s tweets focused on comedy and not on cancer or other medical topics, we excluded this account from our analysis.) A detailed analysis of the remaining 51 accounts was subsequently conducted following their extraction from the dataset.

### Review of the relationships between users

Using the mentionmapp website [21], which enabled us to search for relationships between users on Twitter, we examined the presence and extent of specific relationships between Twitter users. This site graphically displays the number of tweets created most recently by a specific user prior to a search, as well as the relationship that exists between that user and other users (i.e., referring to sending a reply in the form of “@user name” on Twitter one or more times). This secondary search was conducted on December 4, 2011. As the technical capabilities of this Twitter-centric search engine have yet to be clarified by the site’s operators, the period available to send replies that can be detected by a mentionmapp search is unknown.

### Review of user-generated Twitter content

We extracted from our dataset the user account with the greatest number of followers from the accounts of breast cancer patients, who made up the largest population of Twitter users studied here. We subsequently used mentionmapp to extract the Twitter users who had a direct relationship with that primary user. In this way, we were able to extract the user accounts in which a direct relationship was found with the user who had the largest number of followers, as observed by one or more replies being sent. The number of tweets of such accounts per day was analyzed using Whotwi, a tool that displays the number of tweets per day or time zone, as based on an analysis of the most recent 600 tweets of individual accounts [22]. Among these accounts, the account that had the largest number of tweets per day was extracted for further analysis.

The contents of the tweets among the users who tweeted a reply one or more times to the extracted user are described using Bettween, a tool that enables retroactive searching of tweets among users [23]. Furthermore, tweets among cancer patients were also searched in the same manner using the Bettween Search instrument.

The Whotwi and Bettween searches began on December 11, 2011. The Whotwi search was completed this same day, and the Bettween search was carried out over a period of 7 days.

This study was approved by the Ethics Committee at Yamagata University Faculty of Medicine.

## Results

### Characteristics of user accounts

**Figure 3:**
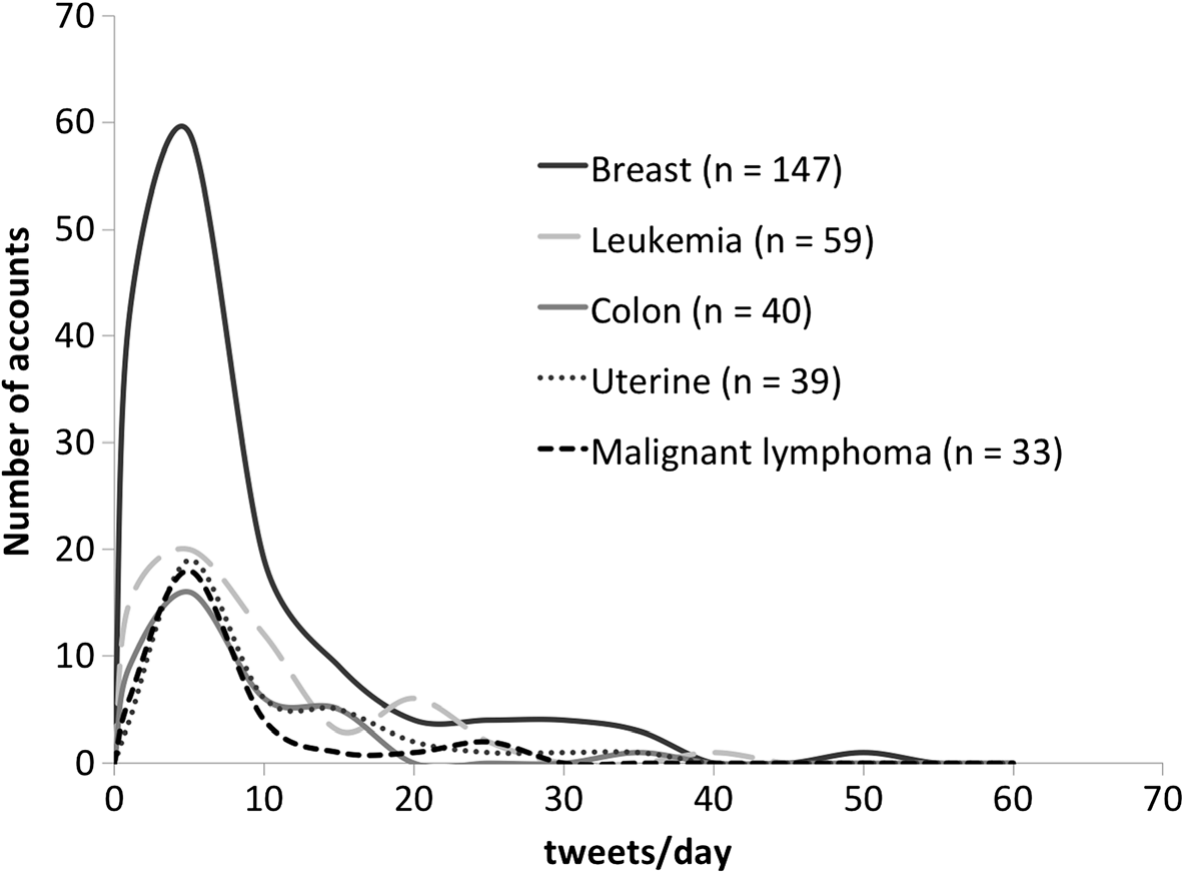
Average number of tweets and number of users per day for Twitter users’ 5 most prevalent types of cancer.

Characteristics of the extracted 51 “power accounts” that had 500 or more followers are shown in Table 1. As previously noted, the term “breast cancer” appeared more frequently than other cancer term in these users’ profiles (n=13). The ratio of males to females in the “power accounts” was 1:1. The Kanto region, which includes the Japanese capital of Tokyo and several other major metropolitan areas, was listed as the home location for almost half of the studied user accounts (n=23). Of the 51 “power accounts,” over half (n=27) of users disclosed their real names, while almost half (n=21) displayed a personal photograph in their profile. The number of tweets per day for the top 5 types of cancer of user accounts is shown in Figure 3. The median of the average number of tweets per day for breast cancer, leukemia, colon cancer, cancers of the uterus and malignant lymphoma was 2.12, 3.79, 3.21, 3.79 and 2.00, respectively, with corresponding ranges of 0.03–14.6, 0.03–16.2, 0.14–13.1, 0.57–22.3 and 0.13–10.7.

**Table 1:** Characteristics of the accounts (followers > 500)

**Variables**		**Numbers**
Sex (male/female/unknown)		24/24/3
Patients (male/female/unknown)	Breast cancer	13 (1/12/0)
	Malignant lymphoma	10 (8/1/1)
	Leukemia	5 (2/3/0)
	Stomach cancer	5 (3/1/1)
	Uterine cancer	4 (0/4/0)
	Brain tumor	4 (4/0/0)
	Colon cancer	4 (2/1/1)
	Renal cancer	1 (1/0/0)
	Prostate cancer	1 (1/0/0)
	Thyroid cancer	1 (0/1/0)
	Lung cancer	1 (1/0/0)
	Bladder cancer	1 (1/0/0)
	Ovarian cancer	10/1/0
		
Area (male/female/unknown)	Hokkaido, Tohoku	1 (1/0/0)
	Kanto	23 (7/14/2)
	Chubu	11 (6/5/0)
	Kinki	8 (4/4/0)
	Chugoku	0
	Shikoku	0
	Kyushu, Okinawa	2 (1/1/0)
	Unknown	6 (5/0/1)
Identified by full name		27 (12/15/0) (52.9%)
Profile photograph of self		21 (11/10/0) (41.2%)
Contained link to any Web site		14 (9/5/0) (27.5%)
Link to own blog		22 (11/10/1) (43.1%)
Followers	Average	2079
	Median	1077
	Minimum	520
	Maximum	33828
		
Tweets	Average	5608
	Median	2370
	Minimum	44
	Maximum	44746
Tweets/day	Average	15.2
	Median	5.7
	Minimum	0.1
	Maximum	126.3

### User connectedness

**Figure 4:**
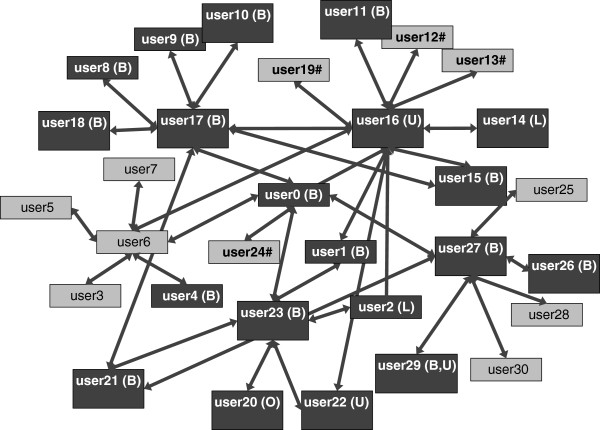
**Relationships between users.** Correlation diagram centered on user0. The users connected by the arrows mutually sent one or more replies. The search was conducted to incorporate friends’ friends. userXX(outlined) : cancer patients. Users who listed their specific type of cancer in their profiles. (B): breast cancer, (L): lung cancer, (O): ovarian cancer, (U): cancer of the uterus. userXX#: Users who are believed to be cancer patients judging from their tweets, although no disease names were described in the profiles (because of descriptions of terms such as anticancer drugs, routine examinations, CT, contrast dyes, bone scintigrams). userXX: Other users.

As previously noted, we opted to exclude from our analysis the account of the Twitter user—a celebrity— who had the largest number of followers; the comedian who owned this account had breast cancer, and her Twitter feed was followed by 33,828 other users. The Twitter account of user0 with the second largest number of followers (2,463 followers) was selected for the previously described December 4, 2011, analysis of the relationship between users. The results of this analysis are shown in Figure 4. The 5 accounts with the most followers all belonged to patients with breast cancer; the remaining 3 accounts from the “Top 5” accounts were those with 1,593, 1,518 and 1,241 followers, respectively.

As shown in Figure 4, it was found that there were cancer patients among the followers of user0. Those followers included 3 breast cancer patients, 1 uterine cancer patient and 1 user who was believed to be a cancer patient. It was found that these cancer patients communicated with one another via tweets, revealing real-life examples of information exchanges among cancer patients via Twitter. Among the 5 “power accounts” with the greatest number of followers, the fourth-largest account also had a network of cancer patients on Twitter (data not shown).

### Content of tweets

The user accounts of cancer patients among the 6 user accounts that had relationships with user0 (5.5 tweets per day) as shown in Figure 4 were these 5 accounts: user16, user17, user23, user24 and user27, showing tweet numbers of 44, 15, 16, unknown and 5.5, respectively, when the search was conducted. Because user24 was set as a non-public user, it was not possible to conduct a search of the user’s tweets.

As a result of our investigation into the contents of the tweets by user16, who had 44 tweets (the largest number) per day, with another 12 users (who were believed to have a relationship with this user, as shown in Figure 4), the contents were classified into categories such as greetings (“good morning,”“good night”); daily conversations or chats (“I did so and so today”); and conversations concerning cancer treatments (“I am going to the hospital today.” The total number of tweets for each category was as follows: 176 for greetings, 139 for daily conversations or chats, and 24 for conversations concerning cancer treatments. The contents of the exchanged tweets about cancer treatments through the network shown in Figure 4 are shown in Table 2. These tweets represented psychological encouragement (12 tweets), greetings when visiting the hospital or reports on the outpatient ward (10 tweets), tweets concerning physical condition (6 tweets) and advice for treatment (2 tweets).

**Table 2:** Conversations regarding treatment*

		
Conversation 1 (psychological encouragement)	user18	I cleared the blood test ♪ but because of a concerning observation above my collarbone (I have had it for 3 years) that I feel has gotten a bit bigger, I had to take an echo test. (>_<)
	user17	Glad to hear that you cleared the test!
	user18	Dear (user17), thank you ♪, now the echo test… Wish me luck(^^)
		
Conversation 2 (psychological encouragement)	user14	Dear (user16), thank you. The medication was effective and I was able to confirm the shrunken CT image. So I think I am ready for chemotherapy. (^O^)/
	user16	Once it turns out to be effective, we feel we'll be able to take it further. Let's do this!!!!!!!!!!!!!!!!!!!!!!!!!!!!!!!!!!!!!!!!!!!!!!!!
Conversation 3 (psychological encouragement)	user16	You don't have to try hard. Just keep yourself in good physical condition for now, so you're ready for the operation next year.After completing treatment, you can come back.
	user16	Dear (user15), keep it up.
	user15	Dear (user16), good morning. (*⌒-⌒*)o ♪ I took a day off from work today.~(¯∇¯;)
	user15	Thank you. (^-^)v I will just take a day off to relax and refresh myself. (*⌒-⌒*)o
	user16	Be careful not to catch a cold.
	user15	Dear (user16), thank you for your kindness as usual. (*´∇`*)
Conversation 4 (report on hospital visit)	user16	Dear (user12), be careful when you visit the hospital.
	user12	Thank you. I am off to the hospital. (*^o^*)
		
Conversation 5 (conversation regarding physical constitution)	user19	Good morning!! I still have some pain 1 week after the operation. Strangely, my left arm which I broke some years ago hurts. Why?
	user16	Because the weather is terrible today, my scar hurts, too.
	user19	Hi sister, good morning! Well, you, too! It's my first time to experience an old wound hurting.Having various pains here and there is confusing (laughing), ha-ha.
	user16	It also hurts just before it starts to rain. Because I have keloid diathesis and my wound is rather wide and mounted, with adhesion, it really hurts when I have intestinal movements. It is really painful when I have diarrhea, but now I am used to it.
	user17	Dear (user19), good morning (^_^). My cut wound from a year ago has been hurting me since yesterday. Although I can bear the pain if I just moan, apparently there are many people who feel pain from old wounds when the weather gets cold. I hate it. Let's keep ourselves warm.
	user19	Dear (user17), good morning. Wow, you, too, dear (user17)! I guess the cold weather does have an effect, after all. Let's keep ourselves warm so that we can heal, everybody. Keep it up today, too.
Conversation 6 (report on hospital visit)	user11	Dear (user16), good evening. Here is your aunt to talk about nice things. (Laughing) It's nice. I feel like drinking tonight… but I will have a gynecological exam tomorrow for the first time in 6 months. Because they will collect my blood as well, I will leave that until tomorrow so I have something to look forward to. d(⌒-⌒)
	user16	Don't miss it.
	user11	I will meet with my favorite attending physician for the first time in 6 months. I'm really looking forward to it. d(⌒-⌒)!
	user16	Me, too. With CT and check-up, there will be two hospital visits this month.
	user11	Just like this year's year-end tax adjustment? For both of us… I will have a gynecological exam tomorrow, too, and the year-end lymph care adjustment the day after tomorrow.(laughing)
Conversation 7 (report on hospital visit)	user11	Dear (user16), good morning. Today is the last lymph care of the year ♪ I am wearing order-made new stockings and feeling great, ready to leave for the doctor's office. d(⌒-⌒)!
	user16	Have a nice day.
Conversation 8 (report on hospital visit)	user16	Dearest (user22), good luck with your bone scintigraphy, RT @(user22): Good morning, everybody, today is the day for the bone scintigraphy~~ .
Conversation 9 (psychological encouragement)	user7	Dearest △△, good morning (^_^)/I totally understand your feelings. Me too, when I was receiving radiotherapy treatment, I really felt depressed whenever I went down the steps, because I felt like I was being told every time "cancer patients are this way?"
Conversation 10 (advice on treatment)	user17	Dearest (user21), good morning. You are now being treated with Xeroda. It's been just a few days, right? Sorry if I am wrong but it may take some time for the drug to take effect.
	user21	Dear (user17), good morning! Oh, Xeroda. Well, if left for 2-and-a-half months without chemotherapy, that seems rational. (´;ω;`) Internal medicine apparently works slowly.(´;ω;`) It will take time, too. (´;ω;`)

*Japanese conversations were translated into English.

## Discussion

This study indicated that Twitter could be a valuable medium for sharing information among cancer patients. A total of 51 Japan-based cancer patients with Twitter accounts were determined by our study to be influential Twitter users as based on their having 500 or more Twitter followers. Although this study examined a considerably smaller sample of influential Twitter users (n=51) than did a previous United States-based study of the “power accounts” of influential tweeting physicians (n=260) [12], our research revealed that cancer patients can empower themselves by tweeting information about their own medical condition and treatment and by providing a forum for the discussion of specific topics.

The breakdown of influential accounts was found to be in the order of breast cancer, leukemia, colon cancer, cancer of the uterus and malignant lymphoma; this differs significantly from the order of cancer prevalence in Japan, in which the top 5 types of cancer are, in descending order: stomach cancer, lung cancer, colon cancer, breast cancer and liver cancer [19]. We found it interesting that the cancer prevalence of our influential users and the general population were so dissimilar. We expect that this discrepancy is associated closely with the widespread Internet usage of the younger population, which made up a disproportionate percentage of our studied Twitter users. Compared with other cancers in our study, breast cancer was seen most in women in their late 30s to early 40s. The Internet usage rate of Japanese women in this age range is as high as 95% [24]; we believe that this high Internet literacy confirms our findings.

Furthermore, while malignant lymphoma or leukemia is a disease with lower numbers of affected people, we found users with these types of cancer to be highly influential in terms of their Twitter connections. This may be a result of the background in which the treatments for leukemia or malignant lymphoma are mainly centered on chemotherapy, with a long treatment period, indicating that treatment for the disease affects the daily life of these patients for a prolonged period. These patients are thus also more likely to have more opportunities over an extended period of time to engage in timely discourse about their individual conditions and treatment.

To better understand how cancer patients influence their followers via Twitter, direct investigation involving the use of a survey of cancer patients with Twitter accounts may be necessary in the future. Examining the distribution of user activities did not reveal any significant differences among the different types of cancer noted in users’ profiles. On the other hand, our study showed that a smaller number of extremely active accounts existed for each type of cancer examined (Figure 3). Under the hypothesis that such small numbers of active users serve as the center of the patients’ networks on social media, we investigated the connections related to the most active users. As a result, we were able to demonstrate that information was exchanged in real time among patients (Figure 4). Based on this finding, we were able to demonstrate for the first time that an information exchange network among patients via social media had already been established.

Of further interest to us is the content of the tweets exchanged among patients. Most of the examined tweets included details of daily life such as greetings or messages concerning treatments, and it was found that almost no medical information concerning cancer was exchanged; this went against our initial expectation that cancer patients would use Twitter to primarily discuss specific cancer-related news and medical information.

Our findings demonstrate that patients use Twitter as a tool of psychological support by being connected among patients, even though it is not a standard or face-to-face method of discussing such information. This observation may support the notion that Twitter plays a unique role that is different from similar-seeming Internet tools such as hospital websites in which patients primarily obtain medical information [2] or blogs in which patients can share their experiences [1]. We expect that as Twitter usage becomes more widespread in the coming years, there will be an attendant rise in the medium’s importance to maintaining—and perhaps improving—public health [25]. However, the dissemination of Twitter among patients in the future may generate various methods of usage, making it necessary to continue careful observation in the future.

Twitter can be used not only with real names but also anonymously, which is often controversial. In our study, 53% of the accounts included the users’ real names, and 41% of the accounts included personal pictures. Investigation into the Twitter accounts of physicians revealed that 78% of these accounts displayed the users’ real names and personal pictures [12], indicating that anonymity is more preferred among patients than physicians. We expect that this discrepancy can be correlated to the fact that information about individuals’ medical conditions is considered personal and confidential, and that revealing a Twitter account user’s name could lead to the disclosure of potentially private medical details. Many people consider it necessary to maintain anonymity when sharing information through Twitter and other social media; such anonymity may be linked to Twitter’s ability to maintain its relevance among the patient populations that use it.

### Limitations

While this study demonstrated that a patient network via Twitter is in the process of being established, there remain several issues to be discussed. First, this study targeted only those Twitter users who described “cancer” either in Japanese Hiragana, Katakana or Kanji letters in their profiles. However, this does not mean that all users who were cancer patients included relevant disease names in their profiles; the absence of cancer details in user profiles could potentially exclude an unknown number of cancer patients from analysis.

Second, because of limitations in search tool performance, we were unable to conduct a large-scale comprehensive qualitative analysis. It is expected that the improvement of search-tool performance will enable larger-scale studies in the future.

Finally, future research into this field of study will need to clarify the types of information most often disseminated via social media. It has been reported that social media often include information that is not necessarily beneficial to the health of media users [26]. Furthermore, Chretien et al. (2011), who studied physicians’ accounts on Twitter, stated that there existed, although rarely, some ethically problematic content, which could possibly violate the patient privacy [12].

Twitter and other forms of social media can prove quite useful in permitting the rapid and timely dissemination of health-related information. However, as social media continue to evolve, they will need to find ways to provide relevant health information without obstructing patient privacy or delivering inappropriate content. Overcoming this point will be an important element in the dissemination of medical information via social media.

## Conclusions

Twitter users with a variety of types of cancer have proved influential on their followers, as demonstrated through the information exchange engaged in by account owners and their followers. Twitter represents a timely and low-cost medium for cancer patients and others seeking information about specific medical conditions, but our study found that the majority of the tweets posted by the 51 users with “power accounts” focused on conversational details (e.g., greetings, cancer treatments) and psychological support rather than the expected medical news and information. Furthermore, Twitter will need to evolve further in order for patients to fully embrace the power of this social medium, as many people are reluctant to reveal personal details via their Twitter accounts. Our study has demonstrated that Twitter is a powerful medium capable of connecting cancer patients via the establishment of a patient network.

## Competing interests

The authors declare that they have no competing interests.

## Authors’ contributions

YS and HN designed the study, provided the study materials, collected and assembled the data and wrote the manuscript. YS, HN, AH, LS, KO and AF analyzed and interpreted the data. All authors reviewed and approved the manuscript.
